# Case Report: Barely Able to Speak, Can’t Stop Echoing: Echolalic Dynamic Aphasia in Progressive Supranuclear Palsy

**DOI:** 10.3389/fnagi.2021.635896

**Published:** 2021-05-04

**Authors:** Marcelo L. Berthier, Florencia Hoet, Álvaro Beltrán-Corbellini, Daniel Santana-Moreno, Lisa Edelkraut, Guadalupe Dávila

**Affiliations:** ^1^Cognitive Neurology and Aphasia Unit, Centro de Investigaciones Médico-Sanitarias, University of Malaga, Málaga, Spain; ^2^Research Laboratory on the Neuroscience of Language, Faculty of Psychology and Speech Therapy, University of Malaga, Málaga, Spain; ^3^Instituto de Investigación Biomédica de Málaga – IBIMA, Málaga, Spain; ^4^Servicio de Otorrinolaringología, Sección Fonoaudiología, Hospital Italiano de Buenos Aires, Buenos Aires, Argentina; ^5^Department of Neurology, Hospital Universitario Ramón y Cajal, Madrid, Spain; ^6^Department of Neurology, Hospital Clinic, Barcelona, Spain; ^7^Area of Psychobiology, Faculty of Psychology and Speech Therapy, University of Malaga, Málaga, Spain

**Keywords:** dynamic aphasia, echolalia, progressive supranuclear palsy, primary progressive aphasia, inhibition deficits

## Abstract

The diagnostic criteria for progressive supranuclear palsy (PSP) incorporate two speech-language disturbances (SLDs), non-fluent/agrammatic primary progressive aphasia and progressive apraxia of speech, but overlook the inclusion of other SLDs, including dynamic aphasia (DA). Thus, there is a need to reappraise the broad spectrum of SLDs in PSP to include other presenting phenotypes. Here we report findings from the study of two elderly patients with PSP presenting with DA and irrepressible echolalia. Both patients had markedly impoverished verbal production, but their performance in other tasks (repetition and naming) and auditory comprehension were preserved or only mildly impaired. Experimental tests of DA revealed impaired word and sentence generation in response to verbal and non-verbal stimuli. Additional language and cognitive testing revealed different types of echolalia (mitigated, automatic, and echoing approval) as well as impaired inhibitory control and social cognition (mentalizing). Both patients had negative neuropsychiatric alterations (i.e., apathy, aspontaneity, and indifference/emotional flatness). Brain magnetic resonance imaging in both patients showed atrophy of the midbrain tegmentum and superior medial frontal cortex suggestive of PSP, yet further evaluation of the neural correlates using multimodal neuroimaging and neuropathological data was not performed. However, based on the already known neural basis of DA and echolalia in PSP and stroke, we suggest that, in the present cases, neurodegeneration in the midbrain tegmentum, superior medial frontal lobe, and caudate nucleus was responsible for DA and that decreased activity in these regions may play a permissive role for eliciting verbal echoing *via* disinhibition of the perisylvian speech-language network.

## Introduction

The clinical features of speech-language disorders (SLDs) in primary progressive aphasias have recently been expanded to include echolalia (Ota et al., 2020), a hitherto forgotten language feature in these disorders (Torres-Prioris and Berthier, 2021). In the same line, the diagnostic criteria for SLDs in progressive supranuclear palsy (PSP) incorporate non-fluent/agrammatic primary progressive aphasia and progressive apraxia of speech (Boxer et al., 2017; Höglinger et al., 2017), yet overlooked alternate language phenotypes [e.g., dynamic aphasia (DA) and echolalia], which can also herald the onset of PSP (Ghika et al., 1995; Esmonde et al., 1996; Della Sala and Spinnler, 1998; Robinson et al., 2006, 2015; Rohrer et al., 2010; Perez et al., 2013; Fernández-Pajarín et al., 2015; Magdalinou et al., 2018). Therefore, it is essential to further delineate the broad spectrum of SLDs in PSP (Catricalà et al., 2019; Peterson et al., 2019) and in other degenerative non-PPA conditions (see Savage et al., 2021). In fact, the language profile of DA (specific deficits in the generation of novel verbal messages) (Luria and Tsvetkova, 1967) in PSP has been clearly delineated (Robinson et al., 2006, 2015), yet less well known is its relationship with concurrent echolalia (repetition of what has been heard) (e.g., Della Sala and Spinnler, 1998; Berthier et al., 2018a). Exploring this association is pertinent because the analysis of language and cognitive deficits in PSP may illuminate the predominant sites of heightened neurodegeneration. Language deficits in non-fluent/agrammatic primary progressive aphasia and progressive apraxia of speech related to PSP point to a predominant left perisylvian neurodegeneration (inferior frontal gyrus and posterior superior temporal gyrus) (Magdalinou et al., 2018) besides the rostral brainstem and basal ganglia involvement. It is also possible that echolalic DA in PSP may additionally involve the midline superior frontal cortex (Kleist, 1960; Ungvari and Rankin, 1990; Berthier, 1999; Rohrer et al., 2010; Perez et al., 2013). This topographical distribution of atrophic changes can account for impoverished speech production in DA, together with echolalia resulting from disinhibition of the mirror neuron system in the frontal and temporoparietal perisylvian cortex (Esmonde et al., 1996; Berthier et al., 2006, 2017, 2018a).

Here we report the findings from the study of two elderly PSP patients initially presenting echolalic DA. To gain further insight into the functional mechanisms underlying these language disorders, we evaluated these two patients with tests tapping language production deficits in DA (Robinson et al., 1998; Berthier et al., 2020). Other tests were also employed evaluating the permissive role of abnormal inhibitory control, social cognition (mentalizing), auditory comprehension, short-term verbal memory, echo awareness, and behavioral changes in the genesis of PSP-related echolalia (Berthier et al., 2017).

## Case Descriptions

According to Movement Disorder Society criteria (Boxer et al., 2017; Höglinger et al., 2017) the two patients described below met the criteria for a diagnosis of *suggestive of PSP* (C1, PSP-SL) and their examination further provided helpful supplementary evidence (dysarthria, dysphagia, and midbrain tegmentum atrophy) that increased diagnostic confidence.

**FIGURE 1:**
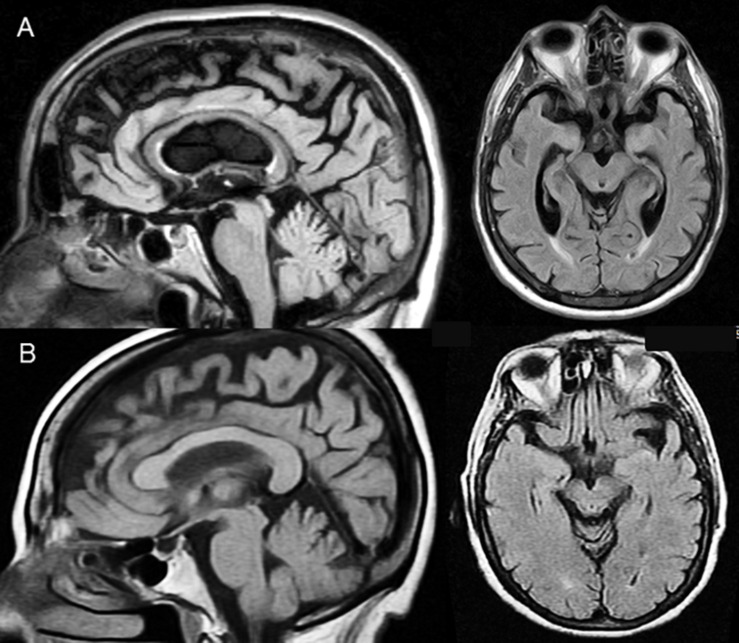
Midsagittal (FLAIR sequences) slices show selective midbrain tegmentum atrophy with “Hummingbird sign” (flattening of the superior aspect of the midbrain tegmentum) in both patients. Axial slices (FLAIR sequences) additionally show a “Mickey Mouse sign” (reduction of the anteroposterior midline midbrain diameter) in patient 1 **(A)** and an incipient “Morning Glory sign” (loss of the lateral convex margin of the midbrain tegmentum) in patient 2 **(B)**, all of which are highly suggestive of PSP. Atrophy is also noted in the superior medial frontal cortex affecting the pre-supplementary motor area and the supplementary motor area, but the cingulate gyrus and the orbitofrontal cortex are preserved. Moderate atrophy of the body of the corpus callosum is seen in patient 1.

Patient 1 was a 71-year-old right-handed man presenting with a 4-year history of progressive decline in speech production. Two years after speech onset of speech production deficits, he suffered two falls and gradually developed motor slowness, difficulty turning over in bed, mild limb rigidity, and micrography. Neurological examination revealed reduced saccades in all directions, bilateral limb rigidity with reduced toe tapping, postural instability, and seborrhea. His family history was positive for Parkinsonism in several members. At the age of 50, his mother developed Parkinsonian symptoms with marked echolalia, eventually evolving into dementia. Two brothers of the patient were diagnosed with Parkinson’s disease, and two maternal female cousins died from Parkinson’s disease (autopsy was not performed). The patient’s verbal production was slow, hesitant, and effortful with reduced phrase length and connective speech. Sentence construction and echolalic emissions occasionally sounded grammatically incorrect. For example, in response to the question “Do you have tremor?” the patient replied “Tremor? No need to have I.” His spontaneous and responsive speech was continuously intermingled with echolalia, and his previously strong regional accent was replaced by a flat intonation devoid of emotional coloring (Berthier et al., 2015). Language initiation was extremely difficult, and the patient needed to stand up and move his right hand to start talking. At rest, he also had right-hand stereotypes. Auditory comprehension, repetition, and naming were preserved. Features consistent with PSP were confirmed with the Progressive Supranuclear Palsy Rating Scale (PSPRS) (Golbe and Ohman-Strickland, 2007; Table 1). A brain magnetic resonance imaging (MRI) showed moderate cortical, subcortical, and midbrain tegmentum atrophy (Figure 1). Treatments with carbidopa/levodopa (25/250 mg/tid) and amantadine (300 mg/bid) were unhelpful.

**TABLE 1:** Progressive supranuclear palsy rating scale (PSPRS).

PSPRS	Patient 1		Patient 2	
	Score	Abnormal exam	Score	Abnormal exam
History	4	Withdrawal, falls (<1 per month), urinary incontinence (few drops)	10	Withdrawal, dysphagia, slow dressing, falls (1–4 per month), and sleep difficulty
Mental exam	3	Bradyphrenia	9	Disorientation, bradyphrenia, emotional incontinence, and grasping behavior
Bulbar exam	1	Dysarthria	1	Dysphagia
Ocular motor exam	5	Voluntary upward, downward, and left and right saccades	2	Slow, hypometric saccades, decreased rate blink
Limb exam	3	Limb rigidity, toe tapping bilaterally	5	Limb rigidity, impaired finger and toe tapping, and tremor
Gait/midline exam	2	Arising from a chair	7	Neck rigidity, arising from a chair, wide-based gait, postural instability, awkward on sitting down
Total score	18		34	

Patient 2 was a 73-year-old right-handed woman with a 5-year history of progressive decline in verbal communication, characterized by sparse and slow speech production. She did not speak unless addressed but produced stereotyped phrases, generated lengthy monologs, while echoing most of what she heard and completing simple open-ended sentences. It was also noticed that her regional accent was reverted to a previous variant learned during childhood (Roth et al., 1997). Her auditory comprehension and naming abilities were mildly impaired, but repetition was virtually intact. Two years after showing first deficits in spoken speech production, she suffered four falls in 1 year and developed apathy, bradyphrenia and emotional incontinence displaying uncontrollable episodes of crying and laughing. Neurological examination disclosed slow, hypometric saccades, decreased rate blink, bilateral limb rigidity with impaired finger and toe tapping, as well as tremor and bilateral hand grasping. She also showed neck rigidity, wide-based gait, postural instability, and problems arising from a chair and shortcomings on sitting down. Features consistent with PSP were revealed with the PSPRS (Golbe and Ohman-Strickland, 2007; Table 1). Family history was negative for movement disorders or dementia. A brain MRI showed moderate cortical, subcortical, and midbrain tegmentum atrophy (Figure 1). Treatments with carbidopa/levodopa (25/250 mg/tid) and amantadine (300 mg/bid) were unhelpful.

The two patients provided written informed consent after receiving a complete description of the study. Written informed consent for publication of any potentially identifiable data or images was also obtained. The Institutional Review Board of the University of Malaga approved this study.

## Testing of Cognition and Language

### Methods

General cognition was evaluated with the Mini Mental State Examination (MMSE) (Folstein et al., 1975), and the Frontal Assessment Battery (FAB) was used to characterize the dysexecutive phenotype (Slachevsky et al., 2004). The profile and severity of aphasia was examined with the Western Aphasia Battery-Revised (WAB-R) (Kertesz and Raven, 2007). Analysis of informativeness in connected speech during picture description of the WAB-R was based on correct information units and related parameters using a rule-based scoring system (Nicholas and Brookshire, 1993). Phonemic fluency and semantic fluency were also evaluated (Borkowski et al., 1967; Kertesz and Raven, 2007).

### Results

Results on cognition and language are shown in Table 2. Scores on the MMSE were normal in patient 1 and moderately impaired in patient 2. Both patients were impaired on the FAB, particularly on verbal fluency and motor series. On the WAB-R, both patients scored in the aphasic range (Aphasia Quotient of the WAB-R ≤ 93.8/100) showing a profile of transcortical motor aphasia (a profile comparable to DA). Analysis of connected speech showed reduced fluency and informativeness as well as pauses and perseverations. Both patients had marked reductions in phonemic and semantic fluencies.

**TABLE 2:** Testing of cognition and language.

	Patient 1	Patient 2
Mini Mental State Examination (max.: 30)	28	21
**Frontal Assessment Battery (FAB)** FAB global score (max. = 18) Similarities (max. = 3) Verbal fluency (max. = 3) Motor series (max. = 3) Conflicting instructions (max. = 3) Go-No-Go (max. = 3) Prehension behavior (max. = 3)	11 2 0 0 3 3 3	8 1 0 0 1 3 3
**Western Aphasia Battery-Revised (WAB-R)** Fluency (max. = 10) Comprehension (max. = 10) Repetition (max. = 10) Naming (max. = 10) Aphasia Quotient (max. = 100) **Analysis of Picture Description (Picnic Scene – WAB-R)** Duration of description (seconds) Word count Total Correct Information Units (CIU) % CIU Perseverations Pauses (>3 s) Phonological fluency (F.A.S.) Semantic fluency (animals)	4 9.0 9.4 8.5 79.8 200 86 65 76 0 14 9 6 (<1% ile)*	4 8.7 9.6 8.6 77.8 40 60 34 57 2 1 2 6 (<1%ile)*

*Norms of healthy controls from Peña-Casanova et al. (2009a).

## Testing of Dynamic Aphasia

The clinical diagnosis of DA was established with the WAB-R (Kertesz and Raven, 2007) on the basis of reduced propositional speech with relative preservation or mild impairment of comprehension, word and sentence repetition, as well as object naming (Luria and Tsvetkova, 1967; Lebrun, 1995). To better identify DA features, several experimental tests were also administered (see next).

### Methods

The characteristics of DA were further evaluated using an adaptation of a series of experimental tests (see Robinson et al., 1998; Berthier et al., 2020). The scores obtained in our PSP patients in each of these tasks were compared to those obtained by a control group (five subjects) using a one-tailed Crawford’s modified *t*-test. This test allows comparing outcomes from one or more individuals with results derived from small control samples (Crawford and Howell, 1998; Crawford and Garthwaite, 2002; Crawford et al., 2010). The following tests were administered: (A) generation of a single word to complete a sentence; (B) generation of a sentence from a single word; (C) generation of a sentence from a given sentence context; (D) generation of a sentence from a single picture; (E) generation of a sentence given a pictorial scene; (F) generation of sentences from a pictorial scene, “what might happen next?”; and (G) story generation from a pictorial context.

### Results

Both patients were impaired in all experimental DA tests (Table 3). The two patients were mildly impaired when a single response was strongly suggested by the sentence frame (high constraint frames in test A), but even stronger impaired in generating words, and phrases when cued by a sentence frame allowing several response possibilities (low constraint frames in test A and tests B and C). They also showed abnormal response generation when the stimuli were non-verbal (pictures or pictorial scenes) (tests D, E, F, and G). It was noteworthy that on performing verbal tests (A, B, and C), both patients always automatically echoed the stimulus sentence first, followed by mitigated echolalia (e.g., test C, stimulus: “My cousin eats apples”; patient’s 1 response: “*apple, your cousin eats apples*”). Automatic echolalia was also frequently heard (e.g., test C, stimulus: “the child paints a flower”; patient 1 response: “*the child paints a flower*”) and several instances of self-contradictory responses (echoing approval). For instance, on replying to the question “Are you tired?” patient 1 replied: “*Yes, I’m not tired!*” Similarly, on completing the open-ended frame of test A: “It’s good to be…,” patient 2 stated “*tired, no! it’s not good to be tired.*” Both patients produced many recurrent verbal perseverations of words and phrases.

**TABLE 3:** Testing of dynamic aphasia with verbal and non-verbal stimuli.

Experimental testing					

	Patient 1	Patient 2	Healthy controls (*n* = 5)	Statistics (Crawford’s t, one tailed)	
				Patient 1	Patient 2
**Verbal tasks**					
Test A. Generation of a single word to complete a sentence					
High constraint frames (max.: 20)	16 (0.80)	17 (0.85)	19.8 ± 0.48	−7.23; *p* < 0.001	−5.32; *p* = 0.003
Low constraint frames (max.: 20)	9 (0.45)	11 (0.55)	19.4 ± 0.89	−10.67; *p* < 0.001	−8.62; *p* < 0.001
Test B. Generation of a sentence from a single word (max.: 20)	11 (0.55)	6 (0.30)	19.4 ± 1.34	−5.72; *p* = 0.002	−9.13; *p* < 0.001
Test C. Generation of a sentence from a given sentence context (max.: 20)	1 (0.05)	5 (0.25)	19.6 ± 0.89	−19.08; *p* < 0.001	−14.97; *p* < 0.001
**Non-verbal tasks**					
Test D. Generation of a sentence from a single picture (max.: 10)	5 (0.50)	6 (0.60)	10 ± 0.0	—*	
Test E. Generation of a sentence given a pictorial scene (max.: 20)	9 (0.45)	4 (0.20)	19 ± 1.73	−5.27; *p* = 0.003	−7.91; *p* < 0.001
Test F. Generation of sentences from a pictorial scene. “what might happen next?” (max.: 20)	0 (0.0)	0 (0.0)	18.75 ± 0.96	−17.83; *p* < 0.001	−17.83; *p* < 0.001
Test G. Story generation from a pictorial context (max.: 10)	1 (0.10)	0 (0.0)	9.8 ± 0.45	−17.85; *p* < 0.001	−19.88; *p* < 0.001

*Test D shows no uncertainty, since standard deviation equals cero.

## Multidimentional Testing of Echolalia

### Methods

The presence of different types of echolalia was elicited in two different contexts: amid a casual conversation and during the administration of WAB-R subtests (spontaneous speech, comprehension, repetition, and naming). The recent literature suggests that other cognitive domains, such as inhibitory control, social cognition (mental state attribution), auditory comprehension, auditory-verbal short-term memory, and awareness, may be dysfunctional in patients with echolalia (Berthier et al., 2017). Therefore, these domains were specifically evaluated in both patients. First, to test inhibitory control, the accuracy and latency in performing the Hayling Sentence Completion Test (HSCT) (Burgess and Shallice, 1996; Pérez-Pérez et al., 2016) were measured through *response initiation* (part A—complete an open-ended sentence with a related word) and *response inhibition* (part B—complete an open-ended sentence with an unrelated word). Moreover, since severe inhibition deficits in part B of the HSCT were evident in both patients (see section “Results” and Table 4), this part was administered using three different strategies to overcome inhibition failures (Robinson et al., 2016). Both patients were informed that after hearing a sentence frame, they needed to look around the room and say aloud the name of an object unrelated to the sentence meaning (strategy 1), or read a number (strategy 2) or a single word (strategy 3) written on a sheet of paper to complete it. Second, to test social cognition, the 10 histories of the Hinting task (HT) were used aimed to infer real intentions behind indirect speech utterances (mentalizing) (Corcoran et al., 1995; Gil et al., 2012). Third, auditory-verbal short-term memory and working memory were respectively evaluated with the forward and backward digit span test of the Wechsler Memory Scale (Wechsler, 2009). Fourth, the comprehension subtest of the WAB-R, resulting from the sum of Yes–No Questions, Auditory Word Recognition, and Sequential Commands subtests, was used to rate auditory comprehension. Finally, an informal interview was also performed to evaluate the patients’ awareness of echolalia and of other disinhibition disorders (hyperlexia and echographia).

**TABLE 4:** Multidimensional Testing of Echolalia.

Tests	Patient 1			Patient 2		
	Accuracy/Proportion	Latency (seconds)		Accuracy/Proportion	Latency (seconds)	
		Correct	Incorrect		Correct	Incorrect
Hayling Sentence Completion Test (HSCT)						
Response initiation (part A) (max. = 15)	13 (0.87)	1.68	4.45	15 (1.0)	2.93	0
Response inhibition (part B) (max. = 15)	0 (0.00)	0	4.52	0 (0.00)	0	1.93
Strategies to Overcome Inhibition Failures						
HSCT – Overt object naming (max. = 15)	9 (0.60)	18.51	9.50	1 (0.06)	1.71	1.57
HSCT – Overt number reading (max. = 15)	14 (0.93)	4.63	4.78	1 (0.06)	1.40	2.81
HSCT – Overt word reading (max. = 15)	11 (0.73)	5.56	3.80	4 (0.27)	4.23	1.78

	**Patient 1**	Controls*		**Patient 2**	Controls*	

Social cognition: Hinting Task (HT)	3	18.03 ± 1.39		5	18.03 ± 1.39	
HT (questions: 2,3,6,7,9)	1	9.41 ± 0.85		2	9.41 ± 0.85	
Auditory-verbal short-term memory						
Digit span						
Forward	4 (3th–5th%ile)**			4 (3th–5th%ile)	Not tested	
Backward	3 (29th–30th%ile)			3 (29th–30th%ile)	Not tested	
Auditory comprehension (WAB-R) (max = 10)	9			8.7		

*Controls from Gil et al. (2012). **Norms of healthy controls from Peña-Casanova et al. (2009b).

### Results

Echolalia was the most outstanding disinhibited behavior in both patients. On analyzing several subtests of the WAB-R, three echolalic subtypes emerged, namely mitigated echolalia (patient 1 = 19; patient 2 = 54), automatic echolalia (patient 1 = 16; patient 2 = 3), and echoing approval (patient 1 = 1; patient 2 = 1) (see the footnote^1^). Occasionally, when patient 1 produced mitigated echolalia, part of the sentence was grammatically incorrect. All these subtypes of echolalia were also frequently observed in other verbal tasks (e.g., experimental DA tests), but no instances of ambient echolalia were heard. The result of the HSCT (accuracy and latency), used to test inhibitory control, is shown in Table 4. Average and perfect performances on the part A (response initiation) were found in patients 1 and 2, respectively, but their performance dropped dramatically on completing part B (response inhibition). To overcome inhibition failures found in part B of the HSCT [0.00], patient 1 used the three strategies [overt object naming (0.60), number reading (0.93), and word reading (0.73)] more efficiently, but at the expense of longer latencies (mean: 9.53 s). However, despite repeated explanations of how to perform the task, inhibition strategies were not useful in patient 2 [overt object naming (0.06), number reading (0.06), and word reading (0.27)]. On the HT, the two patients were markedly impaired in their ability to infer real intentions behind indirect speech utterances (mentalizing) (Table 4). Auditory short-term memory, working memory, and auditory language comprehension were slightly reduced in both patients. The two patients were fully aware of their echolalic behavior to the extent that both commented “I can’t stop repeating what you say,” but they had limited insight into other aspects of their disinhibited behavior (echographia, hyperlexia) (see below; see examples of echolalia in Supplementary Material).

Other disinhibited behaviors were also observed. Patient 1 showed echographia (automatic translation of visual and sometimes auditory stimuli into writing) on spontaneous writing (Pick, 1924; Berthier et al., 2006). Patient 2 showed poor control of inner speech manifested by impulsive figure naming presented during language testing and by describing aloud the actions of people in the room, even though she was not instructed to do so (Tanaka et al., 2000; Vercueil and Klinger, 2001). She commented “I can’t remain silent… I feel obligated to speak and to describe what people do.” She also incurred in long stereotyped monologs at night and occasionally read words impulsively written on commercial signs on the street (hyperlexia) (Suzuki et al., 2009). To unsuccessfully stop her comments, she frequently said, “Now, I shut up.” Neither patient did show utilization behavior for common objects.

## Testing of Behavioral Abnormalities

### Methods

Neuropsychiatric abnormalities are frequent in PSP, particularly apathy, depression, and sleeping problems (Kulisevsky et al., 2001; Gerstenecker et al., 2013). Negative symptoms and disinhibited behaviors were evaluated with the Frontal Behavioral Inventory (FBI) (Kertesz et al., 1997), whereas changes in the frequency and severity of five behaviors (eating and cooking, roaming, speaking, movement, and daily rhythm) were rated with the Stereotypy Rating Inventory (SRI) (Shigenobu et al., 2002). Both inventories were administered to a reliable caregiver.

### Results

Both patients displayed negative symptoms. On the FBI, patient 1 obtained a low negative behavior score (6/36), showing mild changes in items evaluating apathy, indifference/emotional flatness, inflexibility, and comprehension and a moderate change in logopenia, but there were no signs of disinhibited behavior (disinhibition score: 0/36). On the same task, a higher negative behavior score (14/36) was found in patient 2, showing moderate changes in items rating apathy, aspontaneity, indifference/emotional flatness, inflexibility, disorganization, and personal neglect. Her disinhibition score was low (5/36) and pinpointed by perseverations and inappropriateness. On the SRI, patient 1 had stereotyped speaking (say the same things—frequency = 4; severity = 3), movements (right-hand stereotypes, touches persons, collects the same things—frequency = 4; severity = 2), and daily rhythm (fixed routines—frequency = 4; severity = 2). On the same inventory, patient 2 had stereotyped speaking (unable to remain silent, talks what she sees, talk about the same things—frequency = 4; severity = 3) and movements (sits on the same seat—frequency = 4; severity = 2).

## Discussion

The presenting phenotype of SLD in our PSP patients extends the boundaries of the recently developed PPA criteria (Boxer et al., 2017; Höglinger et al., 2017) to include DA. Furthermore, our study expands the phenotype of DA already described in PSP (Esmonde et al., 1996; Robinson et al., 2006, 2015; Magdalinou et al., 2018) by including different types of echolalia coexisting in the same patient (Ghika et al., 1995; Della Sala and Spinnler, 1998; Fernández-Pajarín et al., 2015). The latter finding is one strength of the present study because for the first time we reappraised echolalia using a multidimensional evaluation in an attempt to disentangle the relative contribution of various cognitive deficits underpinning such disinhibited verbal behavior (Berthier et al., 2017, 2018b).

### Dynamic Aphasia

Language features in our patients were consistent with DA since they showed disproportionate deficit in both spontaneous speech and picture description, whereas the production of language in other verbal tests (repetition and naming) as well as auditory comprehension were average or slightly below average. Taking in consideration the characteristics of language and cognitive deficits found in our patients, we classify their DA as belonging to a *domain-general* subtype, a condition secondary to the involvement of frontal and subcortical areas (Robinson et al., 2006). Nevertheless, the production of ungrammatical sentences in both spoken language production and mitigated echolalia in patient 1 also suggests a *language-based* subtype (Robinson et al., 2006) due to involvement of the left anterior perisylvian language cortex (Magdalinou et al., 2018). Both patients showed severe impairments of volition and initiative, key features of DA associated to medial superior frontal lobe damage (Rohrer et al., 2010; Perez et al., 2013). Indeed, patient 1 felt forced to shake his right hand to initiate language production, a barely effective trick that unsuccessfully primed language activation in the left prefrontal cortex (Luria, 1970; Raymer et al., 2002). Similarly, patient 2 never initiated conversations or uttered words spontaneously. However, she impulsively described the actions performed by persons nearby or incurred in endless irrelevant monologs. Further support for the diagnosis of DA comes from the impaired performance of both patients on experimental DA tests, which disclosed widespread verbal generation deficits in response to verbal and non-verbal stimuli in comparison with healthy controls (Robinson et al., 1998, 2006, 2015). Such alterations may reflect the combination of the inability to generate verbal responses, impairments in energization (idea generation) (Barker et al., 2018), and in the generation of a fluent sequence of novel thoughts filled with perseverations (Robinson et al., 2006).

Our present findings suggest that neurodegeneration of the midbrain–basal ganglia–superior medial frontal cortex might be related to impaired discourse generation, thought sequencing, and verbal response selection on experimental DA tests (Magdalinou et al., 2018). The MRIs showed moderate atrophy in the superior medial frontal cortex and the midbrain tegmentum with the typical configuration described in PSP (Mueller et al., 2018) (see Figure 1). The atrophic changes in the pre-supplementary motor area and the supplementary motor area may account for the impairment in controlling shared representations (misunderstanding the intentions of others) (Frith and Frith, 2006; Brass et al., 2009) and evaluating outcomes (e.g., impaired reflection on one’s own performance) (Passingham et al., 2010; Berthier et al., 2018a). Widespread white matter degeneration has been described in PSP (Zhang et al., 2016), and dysfunction of two white matter tracts traveling through the frontal substance may also play a role in language and cognitive deficits and in the behavior in PSP. Damage to the frontal aslant tract, a white matter bundle linking the superior medial frontal cortex with the pars opercularis of the inferior frontal gyrus, may be responsible for impaired verbal fluency and expression recognition of communicative intentions (Catani et al., 2013; Catani and Bambini, 2014). Moreover, the involvement of the frontostriatal tract, which connects the superior medial frontal cortex with the head of the caudate nucleus, could account for impaired initiation and preparation of speech movements and verbal fluency (Kinoshita et al., 2014) as well as of decreased motivation and goal-directed behavior (apathy) (Levy and Dubois, 2006; Lansdall et al., 2017).

### Echolalia and Related Disinhibited Behaviors

On this ground, we also demonstrated that several types of verbal echoing (mitigated echolalia, automatic echolalia, and echoing approval)^2^ occur in PSP-related DA and that they coexist with modality-specific utilization behaviors such as hyperlexia, hypernomia, and echographia (Ghika et al., 1995; Tanaka et al., 2000; Berthier et al., 2006). Altogether, these deficits may reflect a predominant disinhibition of the left perisylvian speech-language network, so that auditory and visual speech perceptions produce hyperexcitability of action-perception circuits including the perisylvian mirror neuron system involved in observation and speech imitation (Watkins et al., 2003; Berthier et al., 2006; Suzuki et al., 2009). There is evidence that modifying the neural activity of the pre-supplementary motor area and the supplementary motor area with non-invasive brain stimulation in healthy subjects induces echophenomena by impairing inhibitory control (Hsu et al., 2011; Finis et al., 2013). In complementary terms, stimulation over the left posterior inferior frontal gyrus facilitates speech repetition (Restle et al., 2012). Therefore, the integration of this information allows us to suggest that automatic activation within the left perisylvian speech-language network resulted from decreased tone in the superior medial frontal cortex and caudate nucleus. This functional uncoupling between areas may account for impaired inhibitory control and social cognition (mental state attribution) as well as for verbal and written echoing (echographia). Awareness of echolalia and related disinhibition activities was variable (preserved for echolalia, absent for hyperlexia and echographia).

One important limitation of our study was that we did not perform structural (voxel-based morphometry and diffusion tensor imaging) and functional neuroimaging (e.g., functional connectivity) necessary to examine the neural mechanisms underpinning echolalic DA. Therefore, further neuroimaging studies and histopathological identification of key areas with heightened neurodegeneration are required. Notice, however, that the main aim of our study was to examine in some detail the cognitive and behavioral mechanisms of DA and echolalia in PSP. Our language and cognitive findings suggest an imbalance between hypoactive midbrain–basal ganglia–superior medial frontal cortex circuits and hyperactive left inferior frontal and temporal superior gyri. To confirm this proposal, future studies may examine a dual dysfunctional mechanism, wherein damage to the midbrain, the superior medial frontal cortex, and the caudate nucleus decreases bottom-up processing causing DA and negative behaviors (apathy). Such damage, in turn, disrupts the top-down modulation of incoming multimodal stimuli in the left perisylvian speech-language network releasing echolalia and other disinhibited behaviors (Berthier et al., 2006).

## Data Availability Statement

The raw data supporting the conclusions of this article will be made available by the authors, without undue reservation.

## Ethics Statement

No animal studies are presented in this manuscript. The studies involving human participants were reviewed and approved by Ethics Committee of the University of Malaga. The patients/participants provided their written informed consent to participate in this study. Written informed consent was obtained from the individuals for the publication of any potentially identifiable images or data included in this article.

## Author Contributions

MB, FH, and GD developed the study concept and the study design. MB, FH, ÁB-C, DS-M, and LE performed testing and data collection. FH, ÁB-C, DS-M, and LE performed the data analysis and interpretation under the supervision of MB and GD. MB and GD drafted the manuscript. All authors provided critical revisions and contributed to the article and approved the submitted version.

## Conflict of Interest

The authors declare that the research was conducted in the absence of any commercial or financial relationships that could be construed as a potential conflict of interest.

## Footnotes

1 Automatic echolalia refers to the immediate production of echoes in an impulsive, “parrot-like” manner. Mitigated echolalia refers to any language change in the echoed emission for communicative purposes Echoing approval refers to the imitation of the affirmative or negative syntactical construction of questions or the intonation (Ghika et al., 1996; Berthier et al., 2018a).

2 Patient 1 also produced instances of mitigated echolalia composed of ungrammatical fragments, an abnormal language feature we termed “agrammatic echolalia.”

## References

[B1] Barker M. S. Nelson N. L. O’Sullivan J. D. Adam R. Robinson G. A. (2018). Energization and spoken language production: evidence from progressive supranuclear palsy. *Neuropsychologia* 119 349–362. 10.1016/j.neuropsychologia.2018.09.004 30195029

[B2] Berthier M. L. (1999). “Transcortical motor aphasia,” in *Transcortical Aphasias*, ed. Berthier M. L. (Hove: Psychology Press), 37–74.

[B3] Berthier M. L. Dávila G. Moreno-Torres I. Beltrán-Corbellini Á Santana-Moreno D. Roé-Vellvé N. (2015). Loss of regional accent after damage to the speech production network. *Front. Hum. Neurosci.* 9:610. 10.3389/fnhum.2015.00610 26594161 PMC4633569

[B4] Berthier M. L. Dávila G. Torres-Prioris M. J. (2018a). “Echophenomena in aphasia: causal mechanisms and clues for intervention,” in *Aphasia Rehabilitation: Clinical Challenges*, eds Coppens P. Patterson J. (Burlington, MA: Jones & Bartlett Learning), 143–172.

[B5] Berthier M. L. Torres-Prioris M. J. López-Barroso D. Thurnhofer-Hemsi K. Paredes-Pacheco J. Roé-Vellvé N. (2018b). Are you a doctor? …Are you a doctor? I’m not a doctor! A reappraisal of mitigated echolalia in aphasia with evaluation of neural correlates and treatment approaches. *Aphasiology* 32 784–813. 10.1080/02687038.2016.1274875

[B6] Berthier M. L. Dávila G. Torres-Prioris M. J. Moreno-Torres I. Clarimón J. Dols-Icardo O. (2020). Developmental dynamic dysphasia: are bilateral brain abnormalities a signature of inefficient neural plasticity? *Front. Hum. Neurosci.* 14:73. 10.3389/fnhum.2020.00073 32265672 PMC7107010

[B7] Berthier M. L. Pulvermüller F. Green C. Higueras C. (2006). Are release phenomena explained by disinhibited mirror neuron circuits? Arnold Pick’s remarks on echographia and their relevance for modern cognitive neuroscience. *Aphasiology* 20 462–480. 10.1080/02687030500484004

[B8] Berthier M. L. Torres-Prioris M. J. López-Barroso D. (2017). Thinking on treating echolalia in aphasia: recommendations and caveats for future research directions. *Front. Hum. Neurosci.* 11:164. 10.3389/fnhum.2017.00164 28420974 PMC5376621

[B9] Borkowski J. G. Benton A. L. Spreen O. (1967). Word fluency and brain damage. *Neuropsychologia* 5 135–140. 10.1016/0028-3932(67)90015-2

[B10] Boxer A. L. Yu J. T. Golbe L. I. Litvan I. Lang A. E. Höglinger G. U. (2017). Advances in progressive supranuclear palsy: new diagnostic criteria, biomarkers, and therapeutic approaches. *Lancet Neurol.* 16 552–563. 10.1016/S1474-4422(17)30157-6 28653647 PMC5802400

[B11] Brass M. Ruby P. Spengler S. (2009). Inhibition of imitative behaviour and social cognition. *Philos. Trans. R. Soc. Lond. B. Biol. Sci.* 364 2359–2367. 10.1098/rstb.2009.0066 19620107 PMC2865080

[B12] Burgess P. W. Shallice T. (1996). Response suppression, initiation and strategy use following frontal lobe lesions. *Neuropsychologia* 34 263–272. 10.1016/0028-3932(95)00104-2 8657357

[B13] Catani M. Bambini V. (2014). A model for social communication and language evolution and development (SCALED). *Curr. Opin. Neurobiol.* 28 165–171. 10.1016/j.conb.2014.07.018 25156623

[B14] Catani M. Mesulam M. M. Jakobsen E. Malik F. Martersteck A. Wieneke C. (2013). A novel frontal pathway underlies verbal fluency in primary progressive aphasia. *Brain* 136(Pt 8), 2619–2628. 10.1093/brain/awt163 23820597 PMC3722349

[B15] Catricalà E. Boschi V. Cuoco S. Galiano F. Picillo M. Gobbi E. (2019). The language profile of progressive supranuclear palsy. *Cortex* 115 294–308. 10.1016/j.cortex.2019.02.013 30884283

[B16] Corcoran R. Mercer G. Frith C. D. (1995). Schizophrenia, symptomatology and social inference: investigating theory of mind in people with schizophrenia. *Schizophr. Res.* 17 5–13. 10.1016/0920-9964(95)00024-G 8541250

[B17] Crawford J. R. Garthwaite P. H. (2002). Investigation of the single case in neuropsychology: confidence limits on the abnormality of test scores and test score differences. *Neuropsychologia* 40 1196–1208. 10.1016/S0028-3932(01)00224-X 11931923

[B18] Crawford J. R. Garthwaite P. H. Porter S. (2010). Point and interval estimates of effect sizes for the case-controls design in neuropsychology: rationale, methods, implementations, and proposed reporting standards. *Cogn. Neuropsychol.* 27 245–260. 10.1080/02643294.2010.513967 20936548

[B19] Crawford J. R. Howell D. C. (1998). Comparing an individual’s test score against norms derived from small samples. *Clin. Neuropsychol.* 12 482–486. 10.1076/clin.12.4.482.7241

[B20] Della Sala S. Spinnler H. (1998). Echolalia in a case of progressive supranuclear palsy. *Neurocase* 4 155–165. 10.1080/13554799808410617

[B21] Esmonde T. Giles E. Xuereb J. Hodges J. (1996). Progressive supranuclear palsy presenting with dynamic aphasia. *J. Neurol. Neurosurg. Psychiatry* 60 403–410. 10.1136/jnnp.60.4.403 8774405 PMC1073893

[B22] Fernández-Pajarín G. Sesar A. Ares-Pensado B. Jiménez-Martín I. Castro A. (2015). Echolalia and progressive supranuclear palsy, an unexpected association. *Rev. Neurol.* 61 143–144. 10.1016/j.nrl.2019.07.004 26178519

[B23] Finis J. Enticott P. G. Pollok B. Münchau A. Schnitzler A. Fitzgerald P. B. (2013). Repetitive transcranial magnetic stimulation of the supplementary motor area induces echophenomena. *Cortex* 49 1978–1982. 10.1016/j.cortex.2012.08.019 23020900

[B24] Folstein M. F. Folstein S. E. McHugh P. R. (1975). “Mini-mental state”. A practical method for grading the cognitive state of patients for the clinician. *J. Psychiatr Res.* 12 189–198. 10.1016/0022-3956(75)90026-6 1202204

[B25] Frith C. D. Frith U. (2006). The neural basis of mentalizing. *Neuron* 50 531–534. 10.1016/j.neuron.2006.05.001 16701204

[B26] Gerstenecker A. Duff K. Mast B. Litvan I. ENGENE-Psp Study Group (2013). Behavioral abnormalities in progressive supranuclear palsy. *Psychiatry Res.* 210 1205–1210. 10.1016/j.psychres.2013.08.045 24035530 PMC3840159

[B27] Ghika J. Bogousslavsky J. Ghika-Schmid F. Regli F. (1996). “Echoing approval”: a new speech disorder. *J. Neurol.* 243 633–637. 10.1002/mds.25103 8892063

[B28] Ghika J. Tennis M. Growdon J. Hoffman E. Johnson K. (1995). Environment-driven responses in progressive supranuclear palsy. *J. Neurol. Sci.* 130 104–111. 10.1016/0022-510x(95)00015-t 7650525

[B29] Gil D. Fernández-Modamio M. Bengochea R. Arrieta M. (2012). Adaptation of the hinting task theory of the mind test to Spanish. *Rev. Psiquiatr. Salud. Ment.* 5 79–88. 10.1016/j.rpsm.2011.11.004 22854578

[B30] Golbe L. I. Ohman-Strickland P. A. (2007). A clinical rating scale for progressive supranuclear palsy. *Brain* 130(Pt 6), 1552–1565. 10.1093/brain/awm032 17405767

[B31] Höglinger G. U. Respondek G. Stamelou M. Kurz C. Josephs K. A. Lang A. E. (2017). Clinical diagnosis of progressive supranuclear palsy: the movement disorder society criteria. *Mov. Disord.* 32 853–864. 10.1002/mds.26987 28467028 PMC5516529

[B32] Hsu T. Y. Tseng L. Y. Yu J. X. Kuo W. J. Hung D. L. Tzeng O. J. (2011). Modulating inhibitory control with direct current stimulation of the superior medial frontal cortex. *Neuroimage* 56 2249–2257. 10.1016/j.neuroimage.2011.03.059 21459149

[B33] Kertesz A. Davidson W. Fox H. (1997). Frontal behavioral inventory: diagnostic criteria for frontal lobe dementia. *Can. J. Neurol. Sci.* 24 29–36. 10.1017/s0317167100021053 9043744

[B34] Kertesz A. Raven J. C. (2007). *The Western Aphasia Battery-Revised.* New York, NY: Psychological Corporation.

[B35] Kinoshita M. de Champfleur N. M. Deverdun J. Moritz-Gasser S. Herbet G. Duffau H. (2014). Role of fronto-striatal tract and frontal aslant tract in movement and speech: an axonal mapping study. *Brain Struct. Funct.* 220 3399–3412. 10.1007/s00429-014-0863-0 25086832

[B36] Kleist K. (1960). Schizophrenic symptoms and cerebral pathology. *J. Ment. Sci.* 106 246–255. 14409774 10.1192/bjp.106.442.246

[B37] Kulisevsky J. Litvan I. Berthier M. L. Pascual-Sedano B. Paulsen J. S. Cummings J. L. (2001). Neuropsychiatric assessment of Gilles de la Tourette patients: comparative study with other hyperkinetic and hypokinetic movement disorders. *Mov. Disord.* 16 1098–1104. 10.1002/mds.1225 11748741

[B38] Lansdall C. J. Coyle-Gilchrist I. T. S. Jones P. S. Vázquez Rodríguez P. Wilcox A. Wehmann E. (2017). Apathy and impulsivity in frontotemporal lobar degeneration syndromes. *Brain* 140 1792–1807. 10.1093/brain/awx101 28486594 PMC5868210

[B39] Lebrun Y. (1995). Luria’s notion of (frontal) dynamic aphasia. *Aphasiology* 9 171–180. 10.1080/02687039508248704

[B40] Levy R. Dubois B. (2006). Apathy and the functional anatomy of the prefrontal cortex-basal ganglia circuits. *Cereb. Cortex* 16 916–928. 10.1093/cercor/bhj043 16207933

[B41] Luria A. R. (1970). *Traumatic Aphasia: Its Syndromes, Psychology, and Treatment.* The Hague: Mouton.

[B42] Luria A. R. Tsvetkova L. S. (1967). Towards the mechanisms of “dynamic aphasia”. *Acta Neurol. Psychiat. Belg* 67 1045–1057. 4972541

[B43] Magdalinou N. K. Golden H. L. Nicholas J. M. Witoonpanich P. Mummery C. J. Morris H. R. (2018). Verbal adynamia in parkinsonian syndromes: behavioral correlates and neuroanatomical substrate. *Neurocase* 24 204–212. 10.1080/13554794.2018.1527368 30293517 PMC6234546

[B44] Mueller C. Hussl A. Krismer F. Heim B. Mahlknecht P. Nocker M. (2018). The diagnostic accuracy of the hummingbird and morning glory sign in patients with neurodegenerative parkinsonism. *Parkinsonism Relat. Disord.* 54 90–94. 10.1016/j.parkreldis.2018.04.005 29643007

[B45] Nicholas L. E. Brookshire R. H. (1993). A system for quantifying the informativeness and efficiency of the connected speech of adults with aphasia. *J. Speech Hear Res.* 36 338–350. 10.1044/jshr.3602.338 8487525

[B46] Ota S. Kanno S. Morita A. Narita W. Kawakami N. Kakinuma K. (2020). Echolalia in patients with primary progressive aphasia. *Eur. J. Neurol.* 28 1113–1122. 10.1111/ene.14673 33305428

[B47] Passingham R. E. Bengtsson S. L. Lau H. C. (2010). Medial frontal cortex: from self-generated action to reflection on one’s own performance. *Trends Cogn. Sci.* 14 16–21. 10.1016/j.tics.2009.11.001 19969501 PMC2806969

[B48] Peña-Casanova J. Quiñones-Úbeda S. Gramunt-Fombuena N. Quintana-Aparicio M. Aguilar M. Badenes D. (2009a). Spanish multicenter normative studies (NEURONORMA Project): norms for verbal fluency tests. *Arch. Clin. Neuropsychol.* 24 395–411. 10.1093/arclin/acp042 19648583

[B49] Peña-Casanova J. Quiñones-Úbeda S. Quintana-Aparicio M. Aguilar M. Badenes D. Molinuevo J. L. (2009b). Spanish multicenter normative studies (NEURONORMA Project): norms for verbal span, visuospatial span, letter and number sequencing, trail making test, and symbol digit modalities test. *Arch. Clin. Neuropsychol.* 24 321–341. 10.1093/arclin/acp038 19661109

[B50] Perez D. L. Dickerson B. C. McGinnis S. M. Sapolsky D. Johnson K. Searl M. (2013). You don’t say: dynamic aphasia, another variant of primary progressive aphasia? *J. Alzheimer Dis.* 34 139–144. 10.3233/JAD-121861 23168447 PMC3621037

[B51] Pérez-Pérez A. MatíasGuiu J. A. Cáceres-Guillén I. Rognoni T. Valles-Salgado M. Fernández-Matarrubia M. (2016). The hayling test: development and normalization of the Spanish version. *Arch. Clin. Neuropsychol.* 31 411–419. 10.1093/arclin/acw027 27246958

[B52] Peterson K. A. Patterson K. Rowe J. B. (2019). Language impairment in progressive supranuclear palsy and corticobasal syndrome. *J. Neurol.* 268 796–809. 10.1007/s00415-019-09463-1 31321513 PMC7914167

[B53] Pick A. (1924). On the pathology of echographia. *Brain* 47 417–429. 10.1093/brain/47.4.417

[B54] Raymer A. M. Rowland L. Haley M. Crosson B. (2002). Nonsymbolic movement training to improve sentence generation in transcortical motor aphasia: a case study. *Aphasiology* 16 493–506.

[B55] Restle J. Murakami T. Ziemann U. (2012). Facilitation of speech repetition accuracy by theta burst stimulation of the left posterior inferior frontal gyrus. *Neuropsychologia* 50 2026–2031. 10.1016/j.neuropsychologia.2012.05.001 22580417

[B56] Robinson G. Blair J. Cipolotti L. (1998). Dynamic aphasia: an inability to select between competing verbal responses? *Brain* 121(Pt 1), 77–89. 10.1093/brain/121.1.77 9549489

[B57] Robinson G. Shallice T. Cipolotti L. (2006). Dynamic aphasia in progressive supranuclear palsy: a deficit in generating a fluent sequence of novel thought. *Neuropsychologia* 44 1344–1360. 10.1016/j.neuropsychologia.2006.01.002 16504225

[B58] Robinson G. A. Spooner D. Harrison W. J. (2015). Frontal dynamic aphasia in progressive supranuclear palsy: distinguishing between generation and fluent sequencing of novel thoughts. *Neuropsychologia* 77 62–75. 10.1016/j.neuropsychologia.2015.08.001 26247320

[B59] Robinson G. A. Walker D. G. Biggs V. Shallice T. (2016). When does a strategy intervention overcome a failure of inhibition? Evidence from two left frontal brain tumour cases. *Cortex* 79 123–129. 10.1016/j.cortex.2016.03.011 27111106

[B60] Rohrer J. D. Paviour D. Bronstein A. M. O’Sullivan S. S. Lees A. Warren J. D. (2010). Progressive supranuclear palsy syndrome presenting as progressive nonfluent aphasia: a neuropsychological and neuroimaging analysis. *Mov. Disord.* 25 179–188. 10.1002/mds.22946 20077483 PMC4608044

[B61] Roth E. J. Fink K. Cherney L. R. Hall K. D. (1997). Reversion to a previously learned foreign accent after stroke. *Arch. Phys. Med. Rehabil.* 78 550–552. 10.1016/s0003-9993(97)90176-3 9161381

[B62] Savage S. A. Suárez-González A. Cassani A. Gopalan R. Stott J. (2021). Non-primary progressive language impairment in neurodegenerative conditions: protocol for a scoping review. *Syst. Rev.* 10:32. 10.1186/s13643-021-01589-6 33472694 PMC7816313

[B63] Shigenobu K. Ikeda M. Fukuhara R. Maki N. Hokoishi K. Nebu A. (2002). The Stereotypy Rating Inventory for frontotemporal lobar degeneration. *Psychiatry Res.* 110 175–187. 10.1016/s0165-1781(02)00094-x 12057829

[B64] Slachevsky A. Villalpando J. M. Sarazin M. Hahn-Barma V. Pillon B. Dubois B. (2004). Frontal assessment battery and differential diagnosis of frontotemporal dementia and Alzheimer disease. *Arch. Neurol.* 61 1104–1107. 10.1001/archneur.61.7.1104 15262742

[B65] Suzuki T. Itoh S. Hayashi M. Kouno M. Takeda K. (2009). Hyperlexia and ambient echolalia in a case of cerebral infarction of the left anterior cingulate cortex and corpus callosum. *Neurocase* 15 384–389. 10.1080/13554790902842037 19585352

[B66] Tanaka Y. Albert M. L. Hara H. Miyashita T. Kotani N. (2000). Forced hyperphasia and environmental dependency syndrome. *J. Neurol. Neurosurg. Psychiatry* 68 224–226. 10.1136/jnnp.68.2.224 10644794 PMC1736777

[B67] Torres-Prioris M. J. Berthier M. L. (2021). Echolalia: paying attention to a forgotten clinical feature of primary progressive aphasia. *Eur. J. Neurol.* 28 1102–1103. 10.1111/ene.14712 33386642

[B68] Ungvari G. S. Rankin J. A. (1990). Speech-prompt catatonia: a case report and review of the literature. *Compr. Psychiatry* 31 56–61. 10.1016/0010-440x(90)90054-v 2404659

[B69] Vercueil L. Klinger H. (2001). Loss of silent reading in frontotemporal dementia: unmasking the inner speech. *J. Neurol. Neurosurg. Psychiatry* 70 705–706. 10.1136/jnnp.70.5.705 11336038 PMC1737330

[B70] Watkins K. E. Strafella A. P. Paus T. (2003). Seeing and hearing speech excites the motor system involved in speech production. *Neuropsychologia* 41 989–994. 10.1016/s0028-3932(02)00316-0 12667534

[B71] Wechsler D. (2009). *Wechsler Memory Scale, Fourth Edition (WMS-IV).* Madrid: Pearson Clinical & Talent Assessment.

[B72] Zhang Y. Walter R. Ng P. Luong P. N. Dutt S. Heuer H. (2016). Progression of microstructural degeneration in progressive supranuclear palsy and corticobasal syndrome: a longitudinal diffusion tensor imaging study. *PLoS One* 11:e0157218. 10.1371/journal.pone.0157218 27310132 PMC4911077

