# Supplementary material for: Case Report: Barely Able to Speak, Can’t Stop Echoing: Echolalic Dynamic Aphasia in Progressive Supranuclear Palsy
Source: Front Aging Neurosci. 2021 May 4;13:635896. doi: 10.3389/fnagi.2021.635896 (PMC8129544; doi:10.3389/fnagi.2021.635896)
Supplement: Supplementary file 1 [file Table_1.DOCX]

**Supplementary Material**

**Echoing approval**

Transcription of Yes-No Questions subtest of the Western Aphasia Battery-Revised

Examiner: - "Is it hot in January?”

Patient 1: - *"Yes. It is not hot in January.”*

Examiner: - "But is it hot in January?”

Patient 1: - *"It is not hot in January.”*

Examiner: - "Is a horse bigger than a dog?”

Patient 1: - *"Yes. A horse is not bigger than a dog."*

Examiner: - "But is a horse bigger than a dog?”

Patient 1: - *"It is not bigger”.*

Examiner: - "Is it smaller than the dog?”

Patient 1: - *"It is smaller. I wish I could say it's bigger than the dog.”*

Examiner: - "But it is not bigger.”

Patient 1: - *"It is not bigger"*

Examiner: - "But let's see JL, a horse is bigger than a dog.”

Patient 1: - *"Yes, it is bigger than a dog."*

Examiner: - "But then, why do you say it's not bigger?"

Patient 1: - *"No... I do say yes."*

Examiner: - "But then we're not clear on that, are we?"

Patient 1: - *"No. A horse is bigger than a dog.”*

**Mitigated echolalia**

Transcription of Yes-No Questions subtest of the Western Aphasia Battery-Revised

Examiner: - "Do you live in Barcelona?

Patient 2: - "No, not Barcelona. I live in Malaga. Yes, in Malaga.

Examiner: - "Are you a woman?

Patient 2: - *"Yes, I am a woman.*

Examiner: - "Are you a doctor?

Patient 2: - *"No, I am not a doctor"*

Patient 2: - "Am I a man?

MT: - *"Yes, you are a man. And my husband is also a man"*

**Descriptions of what others do**

Examiner: - "Is this your house?"

Patient 2: - *"Yes, I think so. But you are gone, and I don't see anyone.*

Examiner: - "I went to close the door."

Patient 2: - *"Yes. To close the door to the corridor."*
